# A 1.15 μW 200 kS/s 10-b Monotonic SAR ADC Using Dual On-Chip Calibrations and Accuracy Enhancement Techniques

**DOI:** 10.3390/s18103486

**Published:** 2018-10-16

**Authors:** Jae-Hun Lee, Dasom Park, Woojin Cho, Huu Nhan Phan, Cong Luong Nguyen, Jong-Wook Lee

**Affiliations:** School of Electronics and Information, Information and Communication System-on-Chip (SoC) Research Center, Kyung Hee University, Yongin 17104, Korea; yijaehun1001@naver.com (J.-H.L.); ektha0203@naver.com (D.P.); chowoojin@khu.ac.kr (W.C.); phnhan2310@gmail.com (H.N.P.) luongnguyen@savarti.com (C.L.N.)

**Keywords:** analog-to-digital converter, successive approximation register, comparator offset, capacitor mismatch calibration

## Abstract

Herein, we present an energy efficient successive-approximation-register (SAR) analog-to-digital converter (ADC) featuring on-chip dual calibration and various accuracy-enhancement techniques. The dual calibration technique is realized in an energy and area-efficient manner for comparator offset calibration (COC) and digital-to-analog converter (DAC) capacitor mismatch calibration. The calibration of common-mode (CM) dependent comparator offset is performed without using separate circuit blocks by reusing the DAC for generating calibration signals. The calibration of the DAC mismatch is efficiently performed by reusing the comparator for delay-based mismatch detection. For accuracy enhancement, we propose new circuit techniques for a comparator, a sampling switch, and a DAC capacitor. An improved dynamic latched comparator is proposed with kick-back suppression and CM dependent offset calibration. An accuracy-enhanced bootstrap sampling switch suppresses the leakage-induced error <180 μV and the sampling error <150 μV. The energy-efficient monotonic switching technique is effectively combined with thermometer coding, which reduces the settling error in the DAC. The ADC is realized using a 0.18 μm complementary metal–oxide–semiconductor (CMOS) process in an area of 0.28 mm^2^. At the sampling rate *f*_S_ = 9 kS/s, the proposed ADC achieves a signal-to-noise and distortion ratio (SNDR) of 55.5 dB and a spurious-free dynamic range (SFDR) of 70.6 dB. The proposed dual calibration technique improves the SFDR by 12.7 dB. Consuming 1.15 μW at *f*_S_ = 200 kS/s, the ADC achieves an SNDR of 55.9 dB and an SFDR of 60.3 dB with a figure-of-merit of 11.4 fJ/conversion-step.

## 1. Introduction

Demand is increasing for various battery-operated sensing systems, for example, the Internet of Things (IoT), which is deployed in various objects for biomedical, home, industrial, and environmental monitoring [1]. For the sensor interface in these applications, very low power consumption is required to provide a long battery lifetime.

To meet the demand, the successive approximation register (SAR) analog-to-digital converter (ADC) has drawn much interest, due to its medium conversion rate and low-power consumption. Various approaches have been proposed to further reduce power consumption. Compared with the conventional structure, the average energy can be reduced by 37.5% using a capacitor splitting technique [2]. When monotonic capacitor switching is used, it saves up to 81.2% [3]. The energy saving is further improved to 87.5% and 96.9% when merged capacitor switching (MCS) and tri-level switching is used, respectively [4,5]. However, the MCS technique demands additional reference voltage with increased circuit complexity. The tri-level switching has a similar drawback, and high energy efficiency is achieved with complex SAR logic.

Monotonic switching uses energy-efficient down switching only. The switching is efficiently realized using a reduced number of switches and capacitors, which simplifies the design of SAR logic [3]. With down switching, the common-mode (CM) voltage at the digital-to-analog converter (DAC) decreases. Therefore, CM-dependent offset calibration is desirable. In a previous work [6], an auto-zeroed comparator is proposed for offset calibration. In reference [7], the offset calibration is performed by using a body terminal that is controlled by a resistive DAC. These works assume a constant CM voltage; therefore, they are not directly applicable to a monotonic SAR ADC.

Mismatch in the capacitive DAC, which occurs due to process variations and routing parasitics, limits the linearity of the ADC. Several techniques have been reported to calibrate the mismatch [7,8,9]. In reference [7], both the comparator offset and mismatch calibrations are applied for a 10-bit split-capacitor ADC; a separate calibrating DAC is used to measure the capacitor mismatch, which consumes additional power and chip area. 

Taking advantage of scaled-down complementary metal–oxide–semiconductor (CMOS) process, various digital calibration techniques have been reported [10,11,12,13]. In reference [11], a low-power calibration technique is presented where circuit blocks, except for the DAC and comparator, are implemented in a field programmable gate array (FPGA). Because the capacitive DAC operates as a single-ended circuit for error evaluation, this approach is rather sensitive to the CM noise. Moreover, the digital calibration usually requires computationally intensive post-processing, and thus, is not suitable for battery-operated low-power sensing applications.

In this paper, we present a low-power SAR ADC realized in an energy and area-efficient manner for a sensor interface. The proposed ADC features (1) fully-integrated on-chip dual calibration and (2) various accuracy-enhancement techniques.

(1) Dual calibration: The proposed calibration techniques, CM-dependent comparator offset calibration (COC) and DAC capacitor mismatch calibration, are realized in an energy and area-efficient manner. By reusing the DAC for generating calibration signals, the CM-dependent comparator offset is calibrated without using separate circuit blocks for calibration. After COC, DAC capacitor calibration is efficiently performed by reusing the comparator for delay-based mismatch detection.
(2) Accuracy-enhancement techniques: To support the calibration operation, we propose new accuracy-enhancement techniques for a comparator, a sampling switch, and a DAC capacitor. We present a dynamic latched comparator, which is robust to kick-back noise. An improved bootstrap sampling switch is proposed, which suppresses a leakage-induced error within 180 μV and a sampling error less than 150 μV. The monotonic switching technique is effectively combined with thermometer coding to reduce the settling error in the DAC.

The measured data indicate the successful operation of the ADC and performance improvement by the proposed dual calibration technique. At a sampling rate of 200 kS/s, the ADC achieves a signal-to-noise and distortion ratio (SNDR) of 55.9 dB and a spurious-free dynamic range (SFDR) of 60.3 dB with a figure-of-merit (FoM) of 11.4 fJ/conversion-step.

## 2. Design

**Figure 1:**
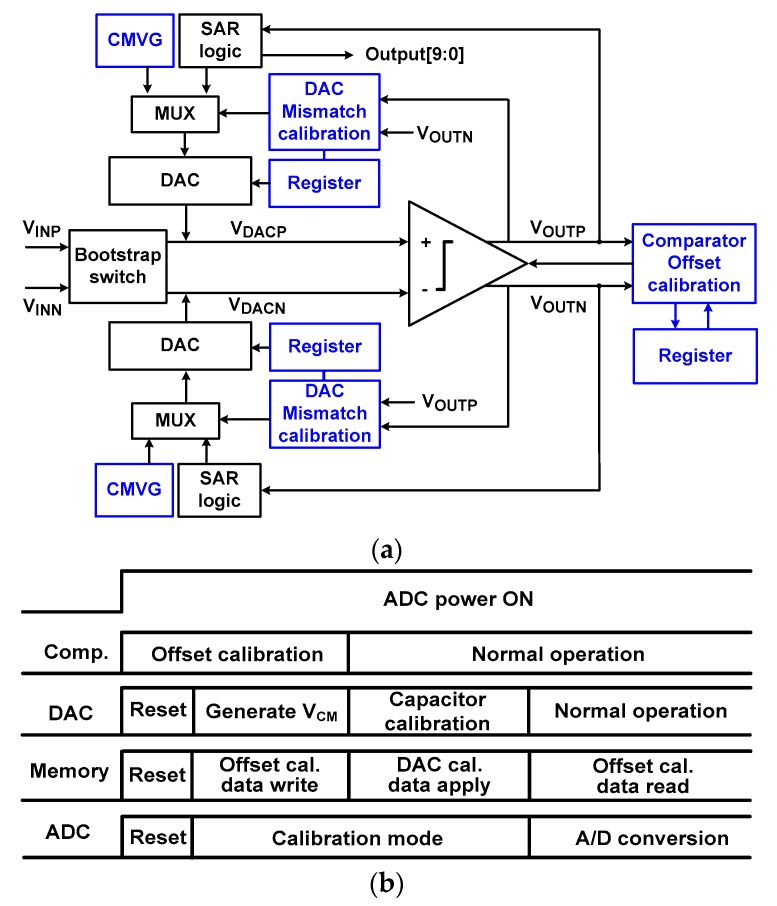
(**a**) Block diagram of the proposed analog-to-digital converter (ADC) with dual calibration. (**b**) Timing sequence of the ADC.

Figure 1a shows a block diagram of the proposed ADC. Top-plate sampling is performed using a bootstrap sampling switch. Monotonic switching is chosen for simple implementation. Thermometer coding is used for the upper 3-bits and binary coding for the remaining 7-bits; the DAC consists of thermometer-coded capacitors C_T_[i] and binary-weighted capacitors C_B_[i] (i = 0 to 6). Thermometer coding reduces the size of the most significant bit (MSB) capacitor from 256 to 64 unit capacitors. Thus, the settling error in the DAC can be reduced.

A straightforward way to reduce the settling error is reserving different delay times for each DAC capacitor [14]. This approach reduces overall conversion time suitable for applications requiring a high conversion rate. The drawback, is that the design of the asynchronous or variable delay logic is rather complicated; for each transition, this approach needs to test a specific condition (DAC voltage settling) to make sure that the previous step is finished before going to the next step. Our work is targeted to sensor applications where a high conversion rate is not needed, but the energy and area efficiencies are important. Although more cycles are needed, the segmented DAC (thermometer + binary coding) reduces differential non-linearity (DNL) errors [15]. In addition, the monotonic switching is efficiently combined with thermometer coding; because monotonic switching performs only down switching, the realization of the thermometer coding is achieved by simply adding shifter registers and switches.

The proposed ADC supports a dual calibration technique for COC and DAC capacitor mismatch calibration. During the COC, we note that the capacitive DAC is not used for analog-to-digital (A/D) conversion. To realize the calibration without using separate circuit blocks, the DAC is reused for implementing the common-mode voltage generators (CMVG). During COC, a multiplexer (MUX) array selects inputs from the CMVG for the DAC. During normal A/D conversion, the inputs from SAR logic are selected. The linearity of SAR ADC is affected by the mismatch in the capacitive DAC. Because high mismatch errors occur for the large capacitors, the DAC mismatch calibration is applied to upper C_T_[i] arrays. To realize the calibration in an energy and area-efficient manner, the lower C_B_[i] are not calibrated; they require sufficient intrinsic linearity. Otherwise, they set the upper limit on performance.

Figure 1b shows the timing sequence for the proposed dual calibration. Without using a separate circuit, the comparator is reused to detect the DAC mismatch. Therefore, COC is performed first and then DAC mismatch calibration follows. The COC includes four steps: reset, offset measurement, writing the calibration data, and applying the calibration during A/D conversion. Similar steps are used for DAC mismatch calibration: reset, mismatch measurement, and applying the calibration data. The data for the DAC mismatch calibration is static and they are applied before A/D conversion. The data for COC are dynamic in nature and they are applied during A/D conversion. During the calibration period, the SAR logic is turned off for power saving.

### 2.1. Dynamic Latched Comparator

The dynamic latched comparator is widely used to reduce power consumption [16]. We consider three issues for the design of the comparator: (1) the clocked operation of the comparator disturbs the top plate of DAC by kick-back; (2) systematic and random device mismatch creates an offset voltage *V*_offset_; (3) during monotonic switching, *V*_offset_ depends on the CM voltage *V*_CM_.

**Figure 2:**
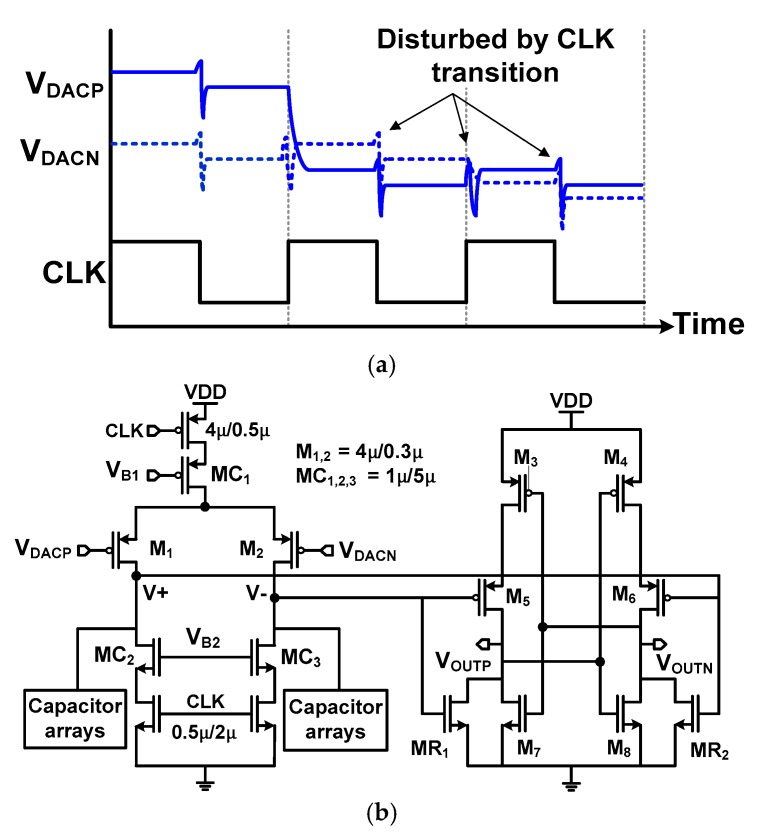
(**a**) Waveform showing the generation of the kick-back noise by the clocked operation. (**b**) Schematic of the comparator using cascode to reduce the kick-back.

Figure 2a shows the waveforms of the DAC voltage *V*_DACP,N_ which are disturbed by the clock transition. In the dynamic latched comparator, the input difference is resolved when the clock signal CLK is switched from low to high. By the CLK transition, the kick-back noise is generated at the input of the comparator by clock feed-through [17]. Then, there is a recovery period when *V*_DACP,N_ settles to a stable voltage. Because this is the time when the comparator starts resolving the input difference, a small asymmetry in this recovery period can cause a decision error.

Figure 2b shows a schematic of the comparator. By the use of auxiliary transistors (MR_1_ and MR_2_), hysteresis can exist in the comparator by the mismatch introduced through the process variations. Therefore, we use the common-centroid layout carefully to suppress the hysteresis. In addition, the comparator is carefully designed for kick-back suppression and CM-dependent offset calibration. To reduce the kick-back, the input transistor pair M_1,2_ is shielded using three cascode transistors MC_1–3_. To increase the output resistance of the MC_1–3_, we choose a small aspect ratio of (W/L) = 1 μm/5 μm so that they operate in the saturation region. By the increased output resistance, the large voltage step created by CLK transition is attenuated as it goes through the MC_1–3_ [18]. The size and bias voltages for the cascode are chosen by circuit simulations. By adjusting the bias voltage *V*_B2_, we are able to control the current through MC_2,3_. When *V*_B2_ is decreased from 1.2 to 0.7 V with *V*_B1_ = 1 V, it effectively reduces the peak current through MC_2,3_ from 7 to 1 μA during the CLK transition. In addition, we perform sizing optimization of M_1,2_ from W/L = 8 μm/0.5 μm to 4 μm/0.3 μm. By the use of cascode and the size optimization, the peak value of kick-back is reduced from 4 to 1 mV. Although the small-size input pair and the cascode reduce the comparator speed, the proposed ADC is targeted for low-speed sensing applications. Therefore, the tradeoff does not greatly affect the overall performance of the ADC.

To handle *V*_offset_, the comparator is calibrated using a binary-weighted capacitor array. Because analog offset calibration requires additional DAC [7], we choose a simple digital approach. Using a register array to store the offset calibration data, CM-dependent offset calibration is performed (See Section B for implementation detail.)

The *V*_offset_ of the comparator consists of static (the first term) and dynamic (the second term) offsets, which can be written as

(1) $$V_{\mathrm{offset}}=\Delta V_{\mathrm{TH}1,2}+\frac{V_{\mathrm{SG}}-\left|V_{\mathrm{TH}1,2}\right|}{2}\left(\frac{\Delta {\left(W/L\right)}_{1,2}}{{\left(W/L\right)}_{1,2}}+\frac{\Delta R_{\mathrm{load}}}{R_{\mathrm{load}}}\right)\;$$

where Δ*V*_TH1,2_ is the threshold mismatch, *V*_TH1,2_ is the threshold voltage, Δ(W/L)_1,2_ is the physical dimension mismatch between M_1_ and M_2_, and Δ*R*_load_ is the load resistance mismatch [3]. The dynamic offset is attributed to charge injection, thus voltage dependent.

**Figure 3:**
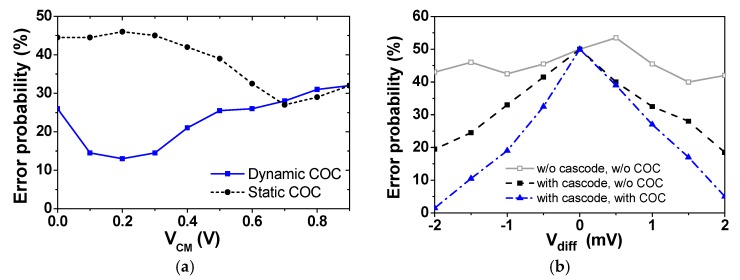
Comparison of error probability as a function of (**a**) common-mode voltage, (**b**) input difference.

Figure 3a compares error probabilities obtained by static and dynamic offset calibrations. The result is obtained using Spectre transient noise simulation with the difference *V*_diff_ = 1 mV applied to the input of the comparator. The sampling rate is *f*s = 4 kS/s and the supply voltage is *V*_DD_ = 1.8 V. The error probability is obtained by counting the case when the comparator makes the wrong decision out of 1000 simulations. The wrong decision is caused by the noise and the *V*_offset_ of the comparator. Because the static approach performs the COC one time at *V*_CM_ = 0.9 V, it reduces the static offset only. The dynamic approach performs the COC at each *V*_CM_ from 0.9 to 0 V with a 112.5 mV step. And this approach reduces both the static and dynamic offsets. The result shows that the two approaches achieve a similar error probability at *V*_CM_ = 0.9 V. In the low *V*_CM_ range, however, the error of dynamic COC is significantly lower than that of static COC.

In addition, we evaluate the error probabilities as a function of *V*_diff_. Figure 3b shows the error probability of three comparators. The result is obtained by performing 1000 Monte Carlo simulations that consider both local and global process variation under a TTT corner. The result confirms that the error is significantly reduced when both cascode and COC are used. In the next section, we describe the implementation details for realizing dynamic COC.

### 2.2. Comparator Offset Calibration

**Figure 4:**
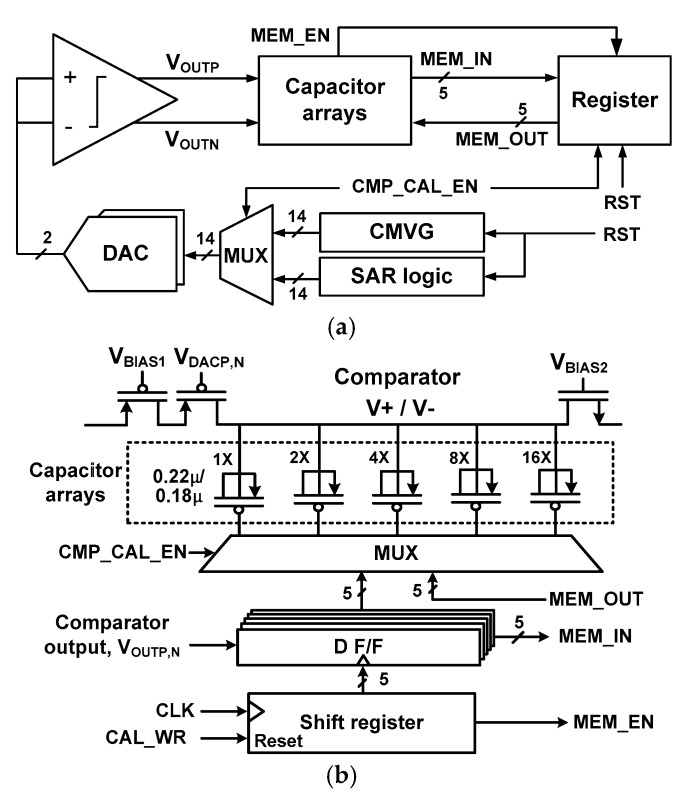
(**a**) Block diagram for comparator offset calibration, (**b**) Control block for the binary-weighted capacitors.

Figure 4a shows a block diagram to implement the dynamic COC. A reset signal RST initializes the digital logic and registers. A MUX controlled by CMP_CAL_EN selects the input to the DAC. When COC is enabled by CMP_CAL_EN = 1, the output from the CMVG is supplied to the DAC. For normal A/D conversion, the output from the SAR logic is input to the DAC.

Figure 4b shows the control block for the binary-weighted capacitor array. The residual offset is in theory reduced by half when increasing the number of calibration bits by one [19]. We determine the number of array elements using circuit simulations. The error probability is reduced by 18.5% and 39.5% when the number of elements is increased to two and five, respectively. Considering the tradeoff between the complexity and the error, we choose 5-bit for the capacitor array elements. The manufacturer’s process specification provides statistical data for the mismatch of the threshold voltage, drain current, and the transconductance as a function of device size ratio (W/L)^0.5^. By using the mismatch data corresponding to the size of the input pair M_1,2_ into (1), we determine a typical *V*_offset_ = 25 mV for the comparator. The proposed offset calibration method allows an offset correction up to ±24 least significant bits (LSB) in the 0.7 LSB step under TTT corner. Although the process corner changes the calibration range up to 20%, the 5-bit calibration scheme still covers the *V*_offset_ range. In addition, the delay of the comparator does not vary significantly with the calibration code. When the mismatch in the DAC capacitor is not considered, it varies from 9 (FFF corner) to 13 ns (SSS corner) with *V*_diff_ = 0.

With the same *V*_CM_ applied to the inputs of the comparator, the state of the capacitor is determined in order of weight by *V*_OUTP,N_. In the case of *V*_OUTP_ = 1 (*V*_OUTN_ = 1), it increases the capacitance at V+ (V−) node. This process is repeated five times. For each *V*_CM_ step, this timing control is performed by the shift register, which is controlled by CAL_WR. The rising edge of CAL_WR clears the D F/F, which holds the previous data for the capacitor array. When the state of 5-bits is determined for a given *V*_CM_, MEM_EN is generated from the last stage of the shift register, which writes the calibration data to the register via MEM_IN[4:0]. This operation repeats until all nine *V*_CM_ steps are processed. During normal A/D conversion, the stored data in the register are sequentially read using MEM_OUT[4:0], which sets the state of the capacitor array.

**Figure 5:**
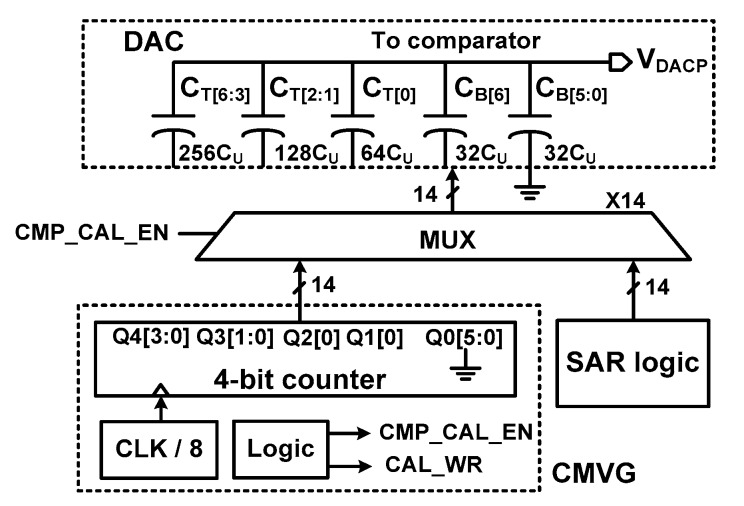
Block diagram of the common-mode voltage generator.

Figure 5 shows the schematic of the CMVG. The CMVG is implemented without using separate circuit blocks by reusing the DAC. To be compatible with monotonic switching where *V*_CM_ is gradually reduced, the CMVG generates nine *V*_CM_ steps of each 112.5 mV in the range from 0.9 to 0 V. Each *V*_CM_ is generated by controlling the bottom plate of the capacitor arrays C_T_[6:0] and C_B_[6:0]. With a reference voltage of 1.8 V, a *V*_CM_ step of 112.5 mV corresponds to 32 *C*_U_ (*C*_U_ = 31.7 fF is a unit capacitor). To complete one cycle of COC, eight clocks are needed; one clock for reset, five clocks for determining the state of the capacitor array, and two clocks for the data store. Therefore, we use a CLK/8 divider for the CMVG. The *V*_DACP,N_ changes its value at every rising edge of CLK/8.

**Figure 6:**
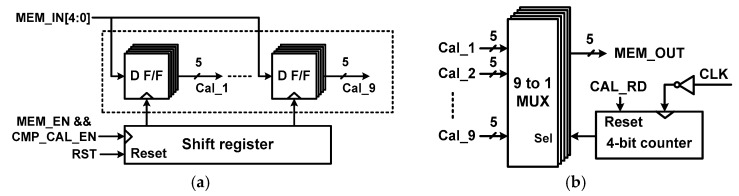
Block diagram of the register control for the (**a**) data write and (**b**) data read operations.

**Figure 7:**
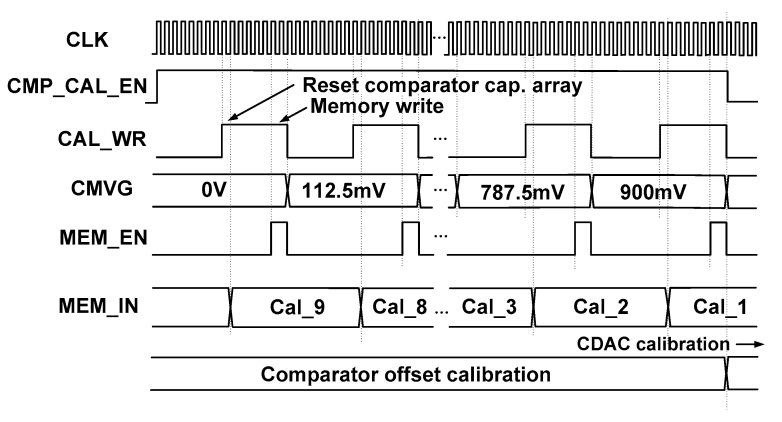
Timing waveform for comparator offset measurement.

Figure 6a shows the block diagram of the register control for writing the calibration data Cal_j[4:0] (j = 1 to 9). When CMP_CAL_EN = 1 and MEM_EN = 1, the output of the shifter register provides the clock for D F/F. Then, MEM_IN[4:0] are written to the register with the rising edge of MEM_EN. During normal A/D conversion, the calibration data stored in the register are sequentially read out using a 9 to 1 MUX and a 4-bit counter as shown in Figure 6b. The calibration data read signal CAL_RD resets a 4-bit counter and starts reading Cal_j[4:0] with every falling edge of CLK. Figure 7 shows the timing waveform for the comparator offset measurement, which starts with CMP_CAL_EN = 1. For each *V*_CM_ step, the rising edge of CAL_WR is used for reset. During the period when CAL_WR = 1, the CMVG generates a *V*_CM_ to determine the state of the capacitor array. Then, the calibration data MEM_IN[4:0] are stored with the MEM_EN signal. When offset measurement for nine *V*_CM_ is finished, CMP_CAL_EN signal becomes low, which indicates the end of comparator offset measurement.

### 2.3. Digital-to-Analo Converter Capacitor Mismatch Calibration

**Figure 8:**
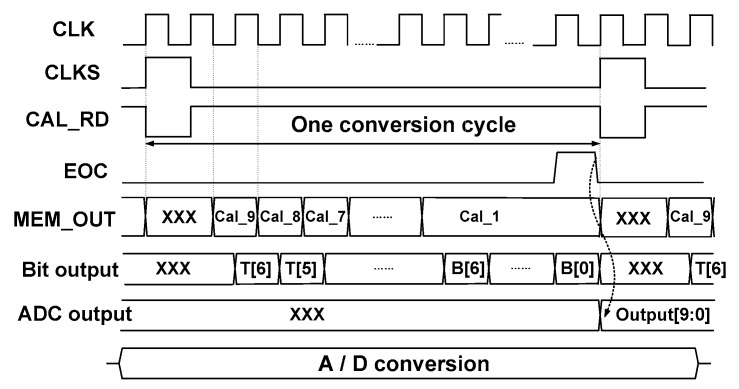
Timing waveform during normal A/D conversion when the offset calibration data are applied.

Figure 8 shows the timing waveform during the normal A/D conversion when the COC is applied. Before the comparator makes a decision, the calibration data MEM_OUT [4:0] are applied to the comparator. When CLK_RD becomes high, MEM_OUT [4:0] are read, which sets the state of the capacitor array in the comparator. When the comparator makes a decision, thermometer-coded bits T[6:0] and binary-coded bits B[6:0] are sequentially generated. When the B[6:0] switches, *V*_CM_ is already close to the ground and does not change significantly. Therefore, Cal_1[4:0] is used during this period. With the end-of-conversion (EOC), the ADC generates outputs.

**Figure 9:**
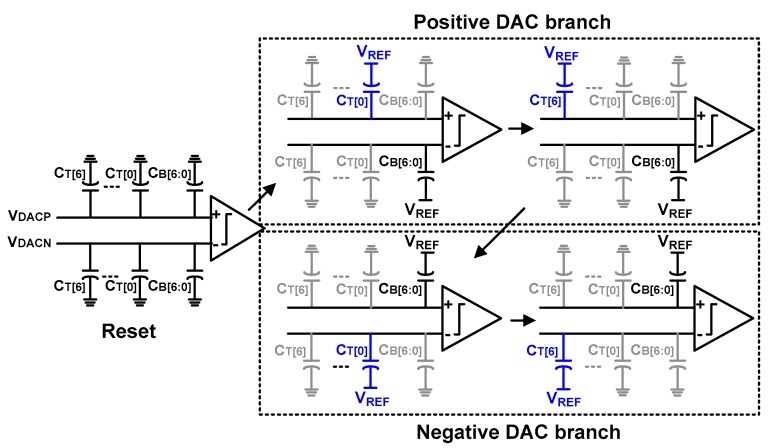
Sequence of the proposed digital-to-analog converter (DAC) mismatch calibration.

Figure 9 shows the sequence of the calibration for detecting the DAC capacitor mismatch. The DAC array consists of C_T_[i] and C_B_[i]. We note that the one-bit of C_T_[i] has a weight of 64*C*_U_. Under the ideal matching condition, the sum of binary capacitors from C_B_[6] to C_B_[0] has the same weight, 64*C*_U_. For the DAC capacitor mismatch calibration, we detect the difference between *V*_DACP_ and *V*_DACN_, which can be used to evaluate the mismatch between the upper and lower DAC. The proposed approach is different from the previous works [7,8,11], which evaluate the mismatch using the DAC in the same branch. Although the previous approach can potentially achieve a better calibration result, it requires a rather complicated calibration logic as well as additional calibration DAC. By reusing the offset-calibrated comparator, our approach evaluates the mismatch in the DAC capacitor without a separate circuit block. A small mismatch in the DAC capacitor leads to a slight difference between *V*_DACP_ and *V*_DACN_, which leads to a large comparator delay for generating output. In the case of a large mismatch, the comparator generates output with a relatively short delay. The delay is encoded using two-bit data for each C_T_[i]. Due to circuit complexity, C_B_[i] is not calibrated.

Using the symmetric properties of the differential structure, the proposed calibration method measures the mismatch between the upper and lower DAC. The mismatch in the one-bit C_T_[i] is sequentially detected by using the sum of C_B_[i] in the other branch. The positive DAC branch is evaluated first and the negative DAC branch is calibrated next. The procedure can be summarized as follows:

(1) Before starting mismatch calibration, the bottom nodes of all capacitors in the DAC are reset by connecting them to the ground.
(2) To evaluate the mismatch error of C_T_[0], connect the bottom plate of C_T_[0] in the positive branch to the reference voltage *V*_REF_ and generate *V*_DACP_. Then, connect the bottom plate of all C_B_[6:0] in the negative branch to *V*_REF_ and generate *V*_DACN_. If there is a mismatch, the difference between *V*_DACP_ and *V*_DACN_ is reflected as the output delay in the comparator.
(3) The delay in the comparator is encoded using two-bit data LSB_P[1:0], which represents mismatch information for C_T_[0] in the positive branch.
(4) Sequentially evaluate the mismatch of the remaining thermometer-coded capacitors (C_T_[1] − C_T_[6]) in the positive DAC branch.
(5) In the same manner, evaluate the mismatch of seven C_T_[i] in the negative DAC branch. This mismatch information is encoded using two-bit data LSB_N[1:0].

**Figure 10:**
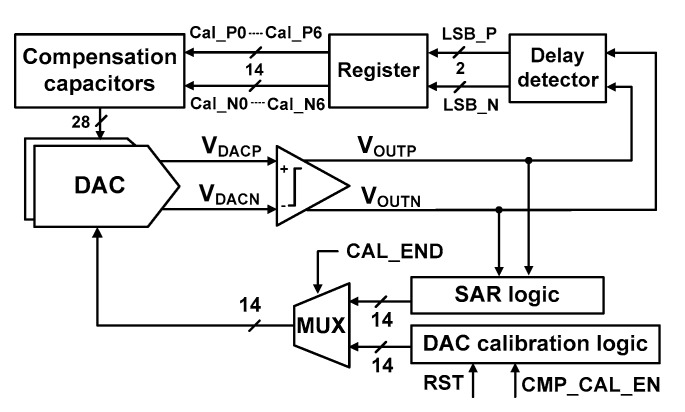
Block diagram for realizing DAC capacitor mismatch calibration.

Figure 10 shows the block diagram for realizing the proposed DAC mismatch calibration. It consists of a DAC calibration logic, a delay detector, registers, and compensation capacitors. During mismatch calibration, CAL_END selects the MUX to receive the input from the DAC calibration logic. The calibration logic sequentially controls the bottom plate of capacitors in the positive and negative DACs. Then, the delay detector generates LSB_P[1:0], which indicates the mismatch data of the positive DAC (LSB_N[1:0] for the negative branch). The two-bit outputs are sequentially written seven times into the register. When the mismatch evaluation is finished for C_T_[0:6], the mismatch data are retrieved from the register. There are seven register outputs for positive (Cal_P0[1:0]-Cal_P6[1:0]) and negative (Cal_N0[1:0]-Cal_N6[1:0]) branches. These outputs are used to set the compensation capacitors attached to each C_T_[0:6].

**Figure 11:**
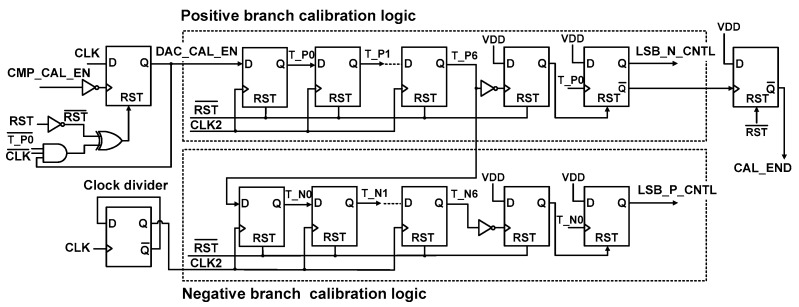
Schematic of the DAC calibration logic.

Figure 11 shows the schematic of DAC calibration logic. It consists of positive/negative branch calibration logic, a clock divider, and a logic gate. Comparator offset and DAC mismatch calibrations are enabled by signals CMP_CAL_EN and DAC_CAL_EN, respectively. The two calibration logics sequentially generate the signals to control the bottom plate of the capacitors in the DAC. To control the other side of the DAC, the period of positive (negative) DAC calibration is set by the LSB_N_CNTL (LSB_P_CNTL) signal. Two clock cycles are used for evaluating the comparator delay and writing the mismatch data to the register, which is generated by the clock divider. From the last stage of D F/F, CAL_END is generated, which indicates the end of the calibration phase.

**Figure 12:**
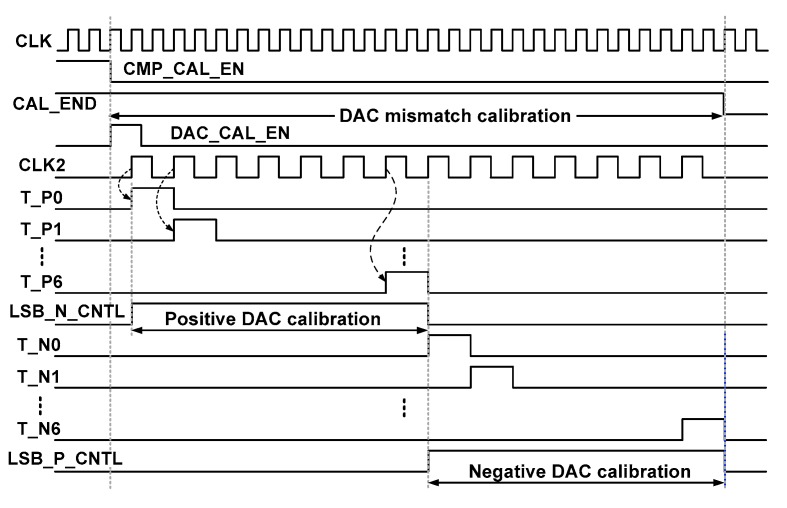
Timing waveform for DAC capacitor mismatch calibration.

Figure 12 shows the timing waveform of the DAC calibration logic. The low-level transition of the CMP_CAL_EN signal indicates the end of COC. Then, the DAC_CAL_EN signal is enabled to perform the DAC capacitor mismatch calibration and the shift registers in the positive branch calibration logic start operation. First, T_P0 becomes active to switch the bottom plate of capacitor C_T_[0] in the positive branch. During this time, LSB_N_CNTL controls the bottom plate of capacitors C_B_[6:0] in the negative branch. The LSB_N_CNTL is enabled until mismatch evaluations are performed for all C_T_[0:6] in the positive DAC branch. The negative branch calibration logic operates in a similar manner using LSB_P_CNTL. When the DAC mismatch calibration is finished, CAL_END becomes low.

**Figure 13:**
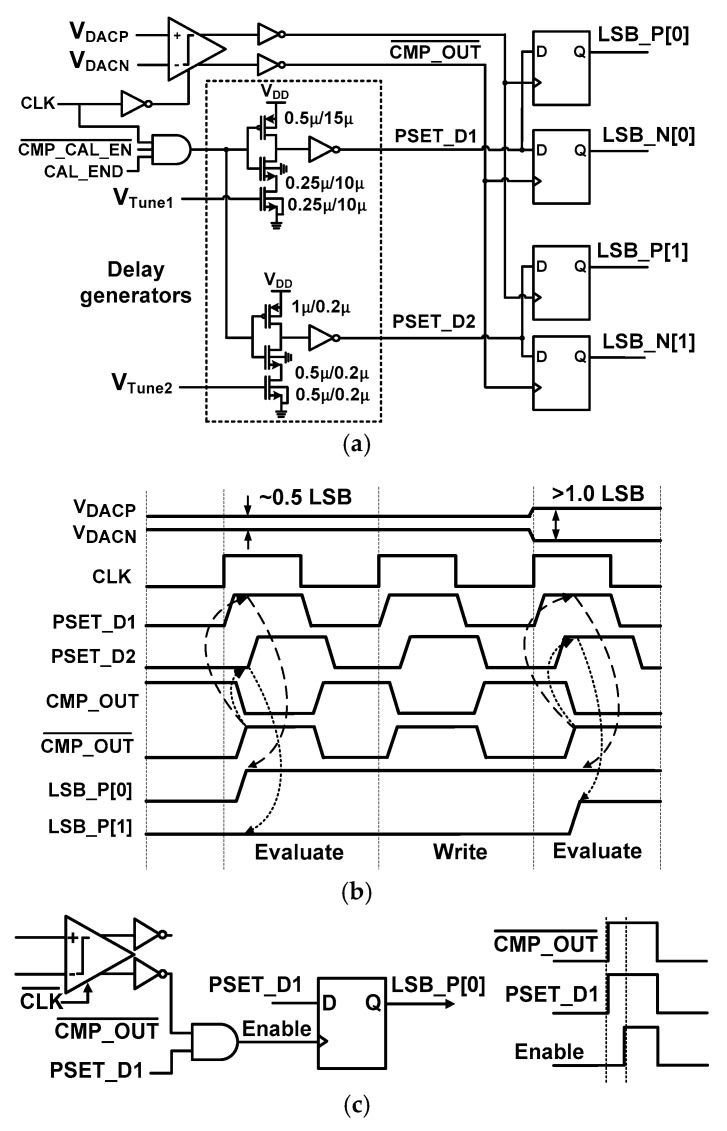
(**a**) Schematic of the delay detector. (**b**) Timing waveform of the delay detector. (**c**) Modified latch control to avoid meta-stability.

Figure 13a shows the schematic of the delay detector. The detector consists of two delay generators and D F/Fs. The outputs PSET_D1,D2 of the delay generator are used for the reference for detecting the mismatch. They are applied to the input terminal of D F/F. Then, the sampling operation of PSET_D1,D2 is performed by the comparator output $\bar{\mathrm{CMP}\_\mathrm{OUT}}$. The sampling evaluates the amount of mismatch existing in the positive (negative) DAC branch using two-bit data LSB_P[1:0] (LSB_N[1:0]).

The comparator delay depends on the DAC capacitor mismatch. When the mismatch error of C_T_[0] in the positive branch is evaluated (See Figure 9), for example, the capacitors in the negative branch is assumed to have sufficient intrinsic linearity; we assume the total sum of these capacitors to be 64*C*_U_ even in the case when the individual capacitor C_B_[i] experiences the worst-case mismatch deviation of 1% from the ideal binary ratio. To meet the requirement, we carefully lay out the routing paths and iteratively trim the size of each capacitor with the aid of a CAD tool. Instead of SAR logic, the DAC calibration logic controls the bottom nodes of capacitors. Then, the difference Δ*V*_in_ between *V*_DACP_ and *V*_DACN_ is obtained [2] using:

(2) $$V_{\mathrm{DACP}}=V_{\mathrm{CM}}+\frac{C_{T}[0]+\Delta C_{T}[0]}{\sum_{i=0}^{6}\left(C_{T}[i\right]+C_{B}[i])+\Delta C_{T}[0]}V_{\mathrm{REF}},\;V_{\mathrm{DACN}}=V_{\mathrm{CM}}+\frac{\sum_{i=0}^{6}C_{B}[i]}{\sum_{i=0}^{6}\left(C_{T}[i\right]+C_{B}[i])}V_{\mathrm{REF}}$$

where ΔC_T_[0] is the deviation from the ideal 64*C*_U_. Similar expressions can be written for C_T_[i]. The delay time *t*_D_ of the comparator can be written as the sum of two terms, the load capacitor discharge time *t*_charge_ and the latch delay time *t*_latch_ [20] as:

(3) $$t_{D}=t_{\mathrm{charge}}+t_{\mathrm{latch}}=2C_{L,\mathrm{out}}\frac{V_{\mathrm{TH}7,8}}{I_{\mathrm{BIAS}2}}+\frac{C_{L,\mathrm{out}}}{g_{m,\mathrm{eff}}}\ln\left(\frac{C_{L,V+}}{C_{L,\mathrm{out}}}\frac{V_{\mathrm{DD}}I_{\mathrm{BIAS}2}^{2}}{8V_{\mathrm{TH}7,8}^{2}g_{\mathrm{mR}1,2}g_{m1,2}\Delta V_{\mathrm{in}}}\right)$$

where *V*_TH7,8_ is the threshold voltage of the transistor M_7,8_ (See Figure 2b), *I*_BIAS2_ is the bias current of the second stage, *C*_L,out_ is the output load capacitance, *C*_L,V+_ is the capacitance at the output of the first stage, *g*_m,eff_ is the effective transconductance of the back-to-back inverter, *g*_mR1,2_ is the transconductance of the intermediate stage transistors MR_1,2_, and *g*_m1,2_ is the transconductance of the input pair. The first term of (3) is independent of Δ*V*_in_ but affected by the process corner. The second term is inversely proportional to Δ*V*_in_.

Using (2), we obtain Δ*V*_in_ of 0.6 and 0.9 LSB for 1.0% and 1.5% capacitor mismatch. Considering some margin for the mismatch, the delay generator is sized to produce proper delay so that mismatch error from 0.5 to 1.5 LSB is detected. To prevent malfunction, we determine the proper delay in the comparator and the delay generator by performing extensive Monte-Carlo simulations. Because the delay in these circuits shares a global process corner, we are able to mitigate the mismatch between delay detectors by using careful layout.

Table 1 shows the delay depending on process corners. The result shows that the comparator delay is reduced when the process corner is changed from SSS to FFF corner as expected. Using the difference between the total delay and the delay without mismatch, we are able to extract the delay depending on the mismatch. Circuit simulations show that the delay has an approximate inverse linear relationship with the error Δ*V*_in_. Therefore, the delay threshold for PSET_D1,D2 is chosen by using three equal delay regions. To deal with the process variation, the delay is further tuned using *V*_TUNE1,2_ in the delay generator.

Figure 13b shows the timing waveform where the mismatch is evaluated in the positive DAC branch. When the mismatch is small, the difference between *V*_DACP_ and *V*_DACN_ is also small, resulting in a relatively long delay in the comparator [21]. For example, consider the case when the mismatch is more than 0.5 LSB but less than 1.0 LSB. In the evaluation phase, $\bar{\mathrm{CMP}\_\mathrm{OUT}}$ rises after PSET_D1 which sets LSB_P[0] to high and LSB_P[1] to low. The LSB_P[0:1] is subsequently written to the register. When the delay detector samples the output of the comparator using D F/F, meta-stability can occur. To remove this, we insert a logic gate to generate an Enable signal as shown in Figure 13c. In this way, the Enable signal provides the clocks for D F/F in a well-defined sequence and removes meta-stability.

**Figure 14:**
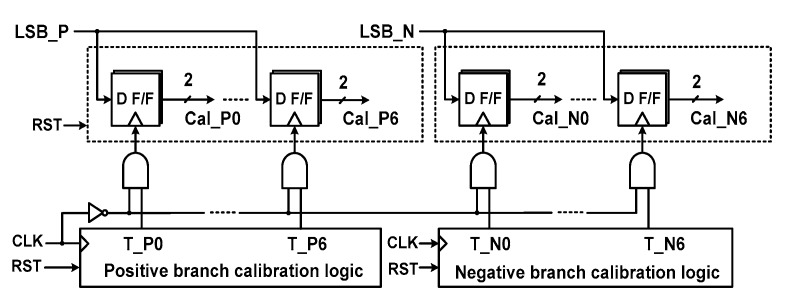
Schematic of the register for storing the calibration data.

**Figure 15:**
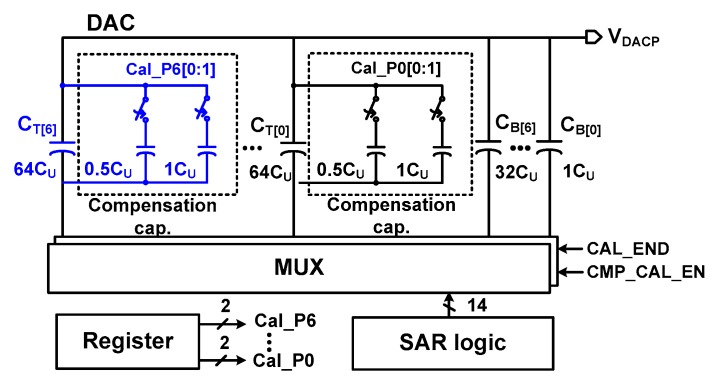
Schematic of compensation capacitors in the positive DAC branch.

By the delay detector, two-bit data (LSB_P[1:0] and LSB_N[1:0]) for each C_T_[0:6] in the two branches are generated. By the calibration logic, the data are sequentially written in the register (Figure 14). The data are used to control the switch for the compensation capacitors attached to each C_T_[0:6], as shown in Figure 15. Each compensation capacitor consists of 0.5*C*_U_ and 1.0*C*_U_. The value of the capacitors is chosen considering a worst-case mismatch (2%) of 64*C*_U_. The effect of calibration can be enhanced by using both add and subtract operations. The subtract operation is implemented by taking advantage of the differential structure [22]. To simplify the logic for the subtract operation and consider the layout parasitic, the size of the original DAC capacitors is reduced by 0.5*C*_U_. Then, the error compensating range is from −0.5 to +1 LSB in the 0.5 LSB step.

In order to assess the performance improvement by the proposed calibration technique, behavioral simulations are performed using Matlab. By including random DAC capacitor mismatch in the behavioral model of the ADC, we perform 1000 Monte-Carlo simulations. Comparator and kT/C noises are not included. The foundry datasheet shows 1% capacitor mismatch which is a conservative estimate. Because there is additional mismatch caused by routing and fringing components, we consider the random mismatch from 1.0% to 2.5%.

**Figure 16:**
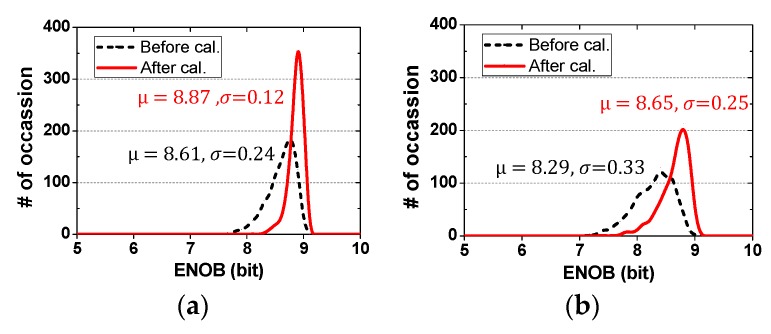

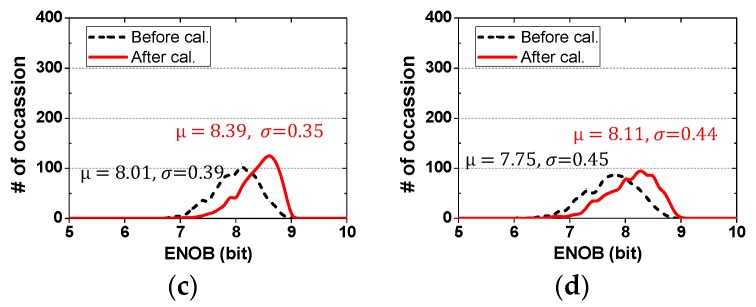
Probability distributions of an effective number of bits (ENOB) before and after DAC capacitor mismatch calibration. Capacitor mismatches are (**a**) 1.0%, (**b**) 1.5%, (**c**) 2.0%, (**d**) 2.5%.

Figure 16 shows the probability distribution of an effective number of bits (ENOB) before and after the mismatch calibration. In the case of 1% mismatch, the average ENOB before and after calibration is 8.61 and 8.87 bits, respectively. The standard deviation is reduced from 0.24 to 0.12 bits by the calibration. In the case of 1.5% mismatch, the average ENOB improves from 8.29 to 8.65 bits. The standard deviation is reduced from 0.33 and 0.25 bits after calibration. The binary-weighted capacitors are not calibrated. With the quantization noise and the discrete value of compensation capacitors, these set the upper limit on performance after calibration. In the case when the binary capacitors are calibrated, the ENOB improves by 0.3–0.4 bits depending on the mismatch. In addition, we perform simulations for static nonlinearity. For 2% mismatch, the peak DNL is +0.09/−0.63 LSB before calibration and it is reduced to +0.08/−0.32 LSB after calibration. The peak integral non-linearity (INL) is +1.71/−1.72 LSB before calibration and it is improved to +0.74/−0.75 LSB after calibration. The result shows that the proposed DAC mismatch calibration is effective at improving both dynamic and static performances of the ADC.

### 2.4. Bootstrap Sampling Switch

**Figure 17:**
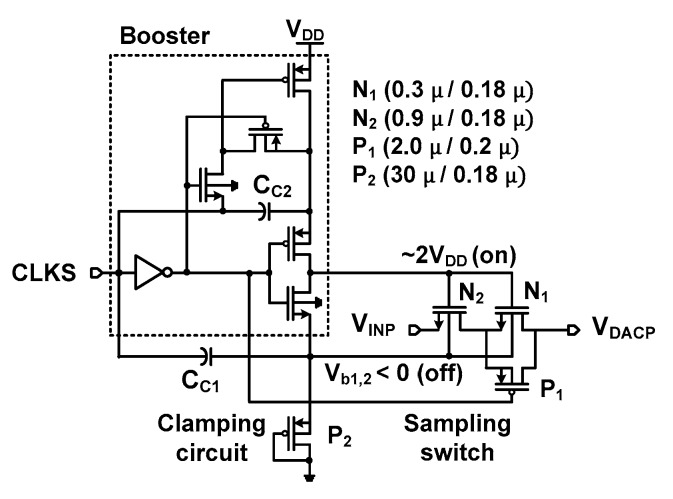
Schematic of the proposed bootstrap sampling switch.

Figure 17 shows the proposed bootstrap sampling switch. Based on the previous work [23], it is modified to reduce leakage-induced error (off-state) and sampling error (on-state). It consists of a booster, a sampling switch, and a clamping circuit.

The sampling error defined by the difference between *V*_DACP_ and *V*_INP_ occurs due to the on-resistance of the switch. To reduce the resistance, a boosted voltage of about 2*V*_DD_ is applied to the gate of N_1_ and N_2_ through the capacitor C_C2_. In addition, P_1_ in parallel with N_1_ forms a transmission-gate which further reduces the on-resistance. To reduce the leakage-induced error, the threshold voltages of N_1_ and N_2_ (both are inside deep *n*-well) are increased by controlling the body terminal. When the sampling clock CLKS is high, the body voltage *V*_b1,2_ is set to the threshold voltage of P_2_ by the clamping circuit. When CLKS goes low, *V*_b1,2_ is reduced by *V*_DD_ through the capacitor C_C1_, which is about −1.3 V. The size of these transistors is optimized by considering the tradeoff between the on-resistance and leakage-induced error.

**Figure 18:**
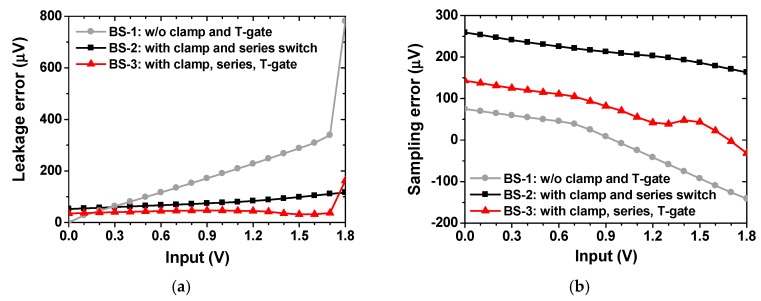
Comparison of three bootstrap sampling switches for (**a**) leakage-induced error and (**b**) sampling error as a function of the input.

Figure 18a compares the leakage-induced error as a function of *V*_INP_. The result is obtained from post-layout simulations with a sampling rate of 3 kS/s. The *V*_DACP_ is measured at 1 ms after the input *V*_INP_ is sampled. Three cases of the bootstrap switch (BS) are shown; (1) BS-1 having transmission-gate without a clamping circuit; (2) BS-2 having a clamping circuit and the series switch (N_1_ and N_2_) only [23]; (3) BS-3 having a clamping circuit and the transmission-gate (Figure 17). In the case of BS-1, the leakage-induced error increases with *V*_INP_ reaching 800 μV at *V*_INP_ = 1.8 V. The result shows that BS-3 has a smaller error than that of BS-2 except at *V*_INP_ = 1.8 V; BS-3 shows a leakage-induced error less than 50 μV up to *V*_INP_ = 1.7 V. The worst-case error is 180 μV. Figure 18b shows the sampling error as a function of *V*_INP_. The turn-on voltage of the sampling switch is 2V_DD_. The gate-to-source voltage *V*_gs_ of the switch, thus, the turn-on resistance depends on *V*_INP_. This causes the sampling error to vary with the input. A constant *V*_gs_ bootstrapping technique is reported [24]; it can be challenging to implement this technique under different process corners. Another work focuses on reducing the turn-on resistance only [25]; this approach can suffer from the leakage error when operated at a low sampling rate. The result shows that the BS-3 shows overall smaller error than that of BS-2; the sampling error is reduced by using a transmission-gate. BS-3 shows a worst-case sampling error of 150 μV, which is higher than that of BS-1. However, the high leakage-induced error of BS-1 is not suitable for our application.

## 3. Measured Results

**Figure 19:**
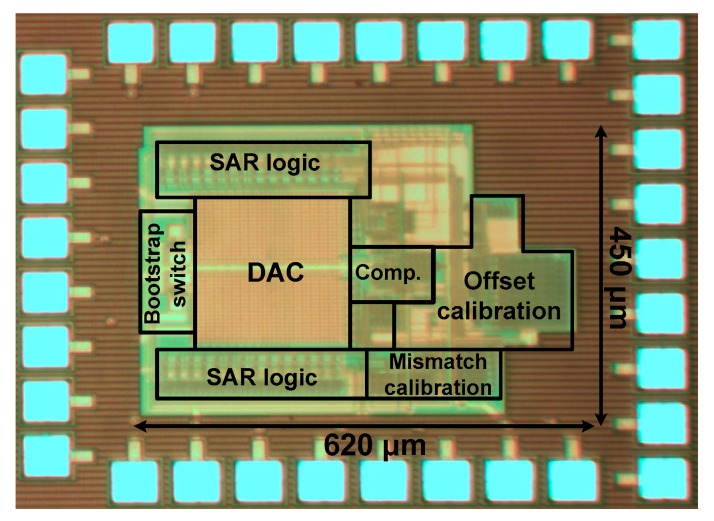
Microphotograph of fabricated ADC.

Figure 19 shows the microphotograph of the ADC fabricated with a 0.18 μm CMOS process. The core area is 0.28 mm^2^.

**Figure 20:**
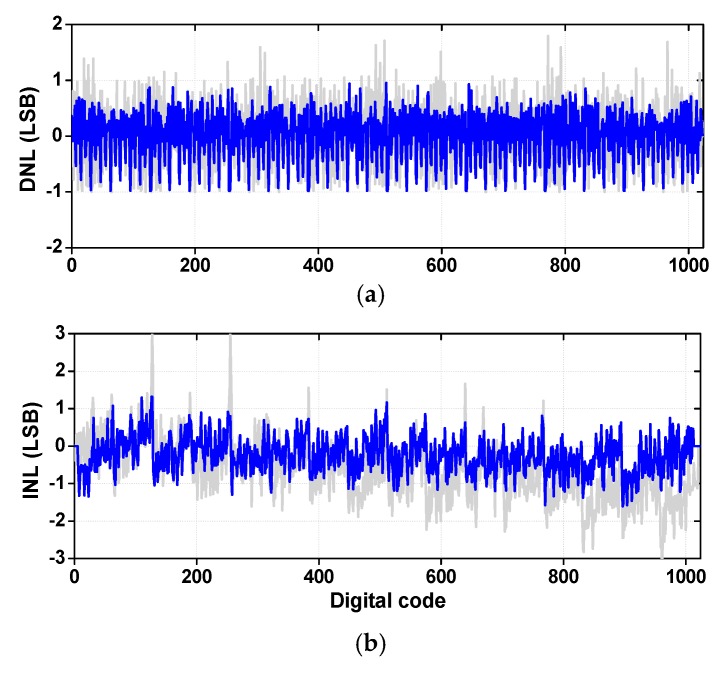
Measured static nonlinearity of ADC. (**a**) differential non-linearity (DNL), (**b**) integral non-linearity (INL).

Figure 20 compares DNL/INL of the ADC before and after COC and DAC capacitor mismatch calibration. A total of 51,200 codes are collected to build a histogram. The peak DNL is +1.79/−1.0 LSB before calibration and it is +0.94/−0.98 LSB after calibration. We note that there is no missing code after calibration. The peak INL is +3.06/−3.17 LSB before calibration and it is reduced to +1.32/−1.61 LSB after calibration.

**Figure 21:**
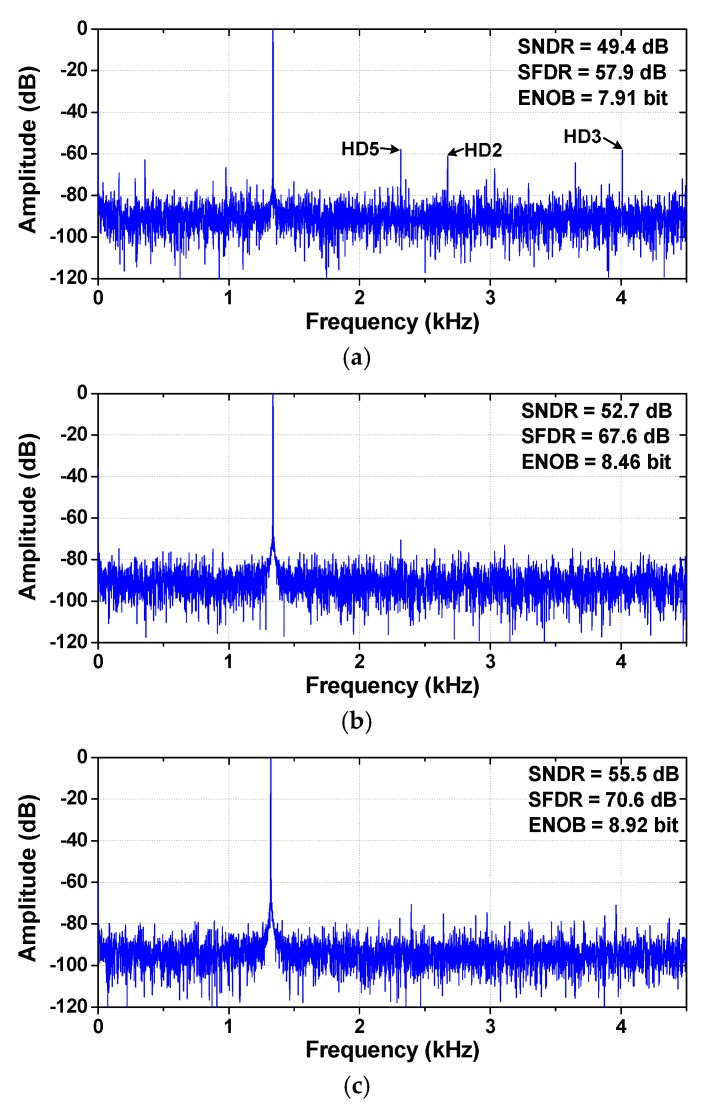
Measured output spectra of the ADC. (**a**) Before calibration, (**b**) after comparator offset calibration, and (**c**) after both comparator offset and DAC capacitor mismatch calibrations. *f*_S_ = 9 kS/s, *f*_IN_ = 1.32 kHz.

Figure 21 compares the measured output spectra of the ADC before and after calibration. The data is obtained from the FFT spectrum with 9000 points. After performing COC, the SNDR and SFDR are improved by 3.3 and 9.7 dB, respectively. When the DAC mismatch calibration is applied, the SNDR and SFDR are improved by 2.8 and 3 dB, respectively. When both COC and DAC calibrations are performed, the SNDR and SFDR are improved to 55.5 and 70.6 dB, respectively, resulting in an ENOB of 8.9 bits.

**Figure 22:**
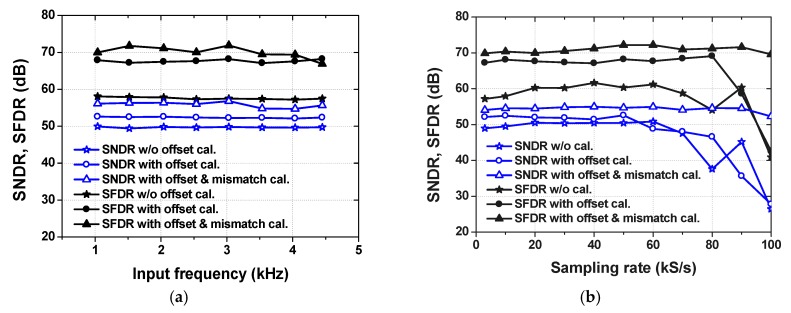
Measured signal-to-noise and distortion ratio (SNDR) and spurious-free dynamic range (SFDR) as a function of (**a**) input frequencies with *f*_S_ = 9 kS/s, (**b**) sampling rates with *f*_IN_ = 1.35 kHz.

Figure 22a shows the measured SNDR and SFDR before and after calibrations as a function of input frequency *f*_IN_. Figure 22b shows the measured SNDR and SFDR before and after calibrations for sampling rate *f*_S_ up to 100 kS/s. We characterize the ADC using a high *f*_IN_.

**Figure 23:**
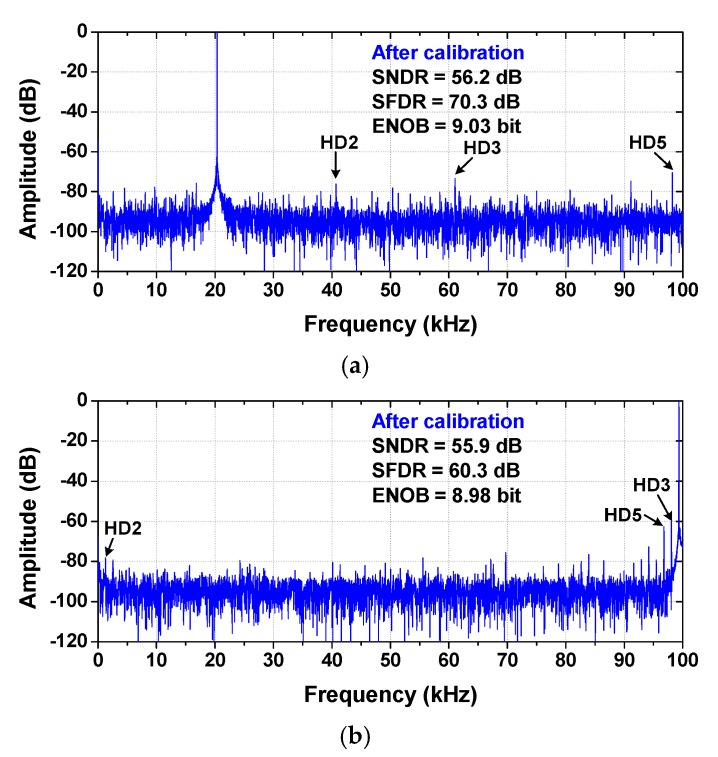
Measured output spectra of the ADC at (**a**) *f*_IN_ = 20.35 kHz, (**b**) *f*_IN_ = 99.35 kHz. *f*_S_ = 200 kS/s.

Figure 23 shows measured spectra of the ADC for *f*_IN_ = 20.35 kHz. When both COC and DAC calibration are performed, the measured SNDR and SFDR are 56.2 and 70.3 dB, respectively, resulting in an ENOB of 9.0 bits. At the Nyquist frequency, the measured SNDR and SFDR are 55.9 and 60.3 dB, respectively.

**Figure 24:**
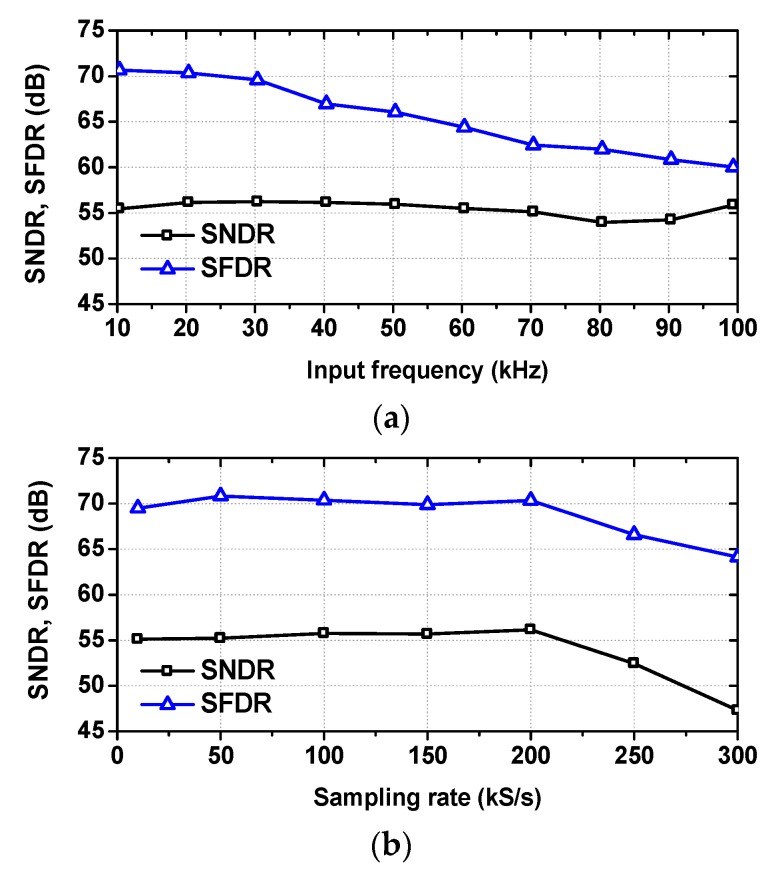
(**a**) Measured SNDR and SFDR at different input frequencies. *f*_S_ = 200 kS/s, (**b**) Measured SNDR and SFDR at different sampling rates. *f*_IN_ = 4.35 kHz.

Figure 24a shows measured SNDR and SFDR as a function of *f*_IN_. The SFDR decreases with *f*_IN_ and the SNDR remains relatively constant up to 100 kHz. Figure 24b shows measured SNDR and SFDR as a function of *f*_S_. Both SNDR and SFDR remain relatively constant up to *f*_S_ = 200 kS/s.

The overall power consumption of the ADC is 1.15 μW. The power breakdown shows that the DAC, the comparator, SAR logic, and calibration blocks consume 101 (8.8%), 516 (44.9%), 280 (24.3%), and 253 nW (22%), respectively. Comparison with the other works is shown in Table 2.

The work in reference [11] presents a low-power (~0.7 μW) SAR ADC for which calibration is performed off-chip. The work in [27] presents a 13-bit SAR ADC with on-chip calibration in a relatively large area of 0.9 mm^2^ using 0.13 μm CMOS. Our work is realized using 0.18 μm CMOS in a compact chip area of 0.28 mm^2^. Both the works [28,29] show relatively low-power consumption, however, it is achieved with relatively low *f*_S_ = 20 and 1 kS/s, respectively. To capture all these tradeoffs, we can use the figure-of-merit (FoM), which is defined as:

(4) $$\mathrm{FoM}=\frac{\mathrm{Power}}{f_{S}\times 2^{\mathrm{ENOB}}}=\frac{\mathrm{Power}}{2\times \mathrm{ERBW}\times 2^{\mathrm{ENOB}}}$$

where effective resolution bandwidth (ERBW) is approximately equal to half of the sampling frequency. The work in [26] shows a good FoM of 8.0 fJ/conv-step using a bypass window, however, it requires a fine-tuned reference voltage to accurately set the bypass window. Consuming 1.15 μW at *f*_S_ = 200 kS/s, the ADC in this work achieves a good FoM of 11.4 fJ/conv-step.

## 4. Conclusions

We present a low-power SAR ADC with dual on-chip calibration technique applied for COC and DAC capacitor mismatch correction. The proposed calibration technique is realized in an energy and area-efficient method that does not use separate circuit blocks. In addition, various accuracy-enhancement techniques further improve the performance of the ADC. A monotonic switching technique is efficiently combined with thermometer coding to reduce the error caused by incomplete settling. The CM-dependent comparator offset is dynamically calibrated by reusing the differential DAC. In addition, the dynamic latched comparator is carefully designed to remove decision errors due to kick-back noise. The evaluation of the DAC capacitor mismatch is performed by reusing the comparator for delay measurement. The calibration of the DAC mismatch in the thermometer-coded seven MSB bits is efficiently performed by using the symmetry property of the differential structure. Measured data show the successful operation of the proposed dual calibration technique. At *f*_S_ = 9 kS/s, the proposed ADC achieves the measured SNDR of 55.5 dB and the SFDR of 70.6 dB. At an increased *f*_S_ = 200 kS/s, the ADC achieves an SNDR of 55.9 dB and an SFDR of 60.3 dB with a FoM of 11.4 fJ/conversion-step. The results indicate the potential of our work for low-power sensing applications.

## Figures and Tables

**Table 1:** Delays depending on process corner and tuning voltages.

Process Corner		SSS	TTT	FFF
CMP_OUT (ns)	Total delay *	21	17	13
	Delay w/o mismatch **	13	11	9
	Delay by the mismatch	8	6	4
PSET_D1 (ns)		2.7	2	1.3
PSET_D2 (ns)		5.4	4	2.6
*V*_TUNE1_, *V*_TUNE2_ (mV)		543, 539	496, 492	420, 414

* The delay range is obtained for the capacitor mismatch corresponding to the Δ*V*_in_ from 0.5 to 1.5 LSB. ** This delay range is mainly from capacitor discharge time with Δ*V*_in_ = 0. SSS (slow NMOS, slow PMOS, slow Poly), TTT (typical NMOS, typical PMOS, typical Poly), FFF (fast NMOS, fast PMOS, fast Poly)

**Table 2:** Comparison with other works.

	[11]	[26]	[27]	[28]	[29]	This Work
Technology (nm)	130	180	130	65	130	180
Supply voltage (V)	0.5	0.6	0.5	0.55	1.0/0.4	1.8/1.0
Resolution (bit)	11	10	13	10	10	10
Sampling rate (kS/s)	10	200	40	20	1	200
SNDR (dB)	61.8	57.5	66.3	55.0	56.7	55.9
SFDR (dB)	77.5	66.7	71.0	68.8	67.6	60.3
ENOB * (bit) @Nyquist	9.93	9.26 ^†^	10.7	8.84	9.1	8.98
Calibration	Off-chip	-	On-chip	-	-	On-chip
Core area (mm^2^)	0.58	0.08	0.90	0.21	0.19	0.28
Power (μW)	0.73 ^††^	1.04	1.47	0.21	0.05	1.15
FoM (fJ/conv-step)	74.8	8.0	17.9	22.4	94.5	11.4

^†^ Input frequency of 20 kHz. ^††^ Not including the power consumption of FPGA. * ENOB = (SNDR − 1.76)/6.02.

## References

[1] Khan M.S., Islam M.S., Deng H. (2014). Design of a reconfigurable RFID sensing tag as a generic sensing platform toward the future internet of things. IEEE Internet Things J..

[2] Ginsburg B., Chandrakasen A. An energy efficient charge recycling approach for a SAR converter with capacitive DAC. Proceedings of the 2005 IEEE International Symposium on Circuits and Systems.

[3] Liu C., Chang S., Huang G., Lin Y. (2010). A 10-bit 50-MS/s SAR ADC with a monotonic capacitor switching procedure. IEEE J. Solid-State Circuits.

[4] Hariprasath V., Guerber J., Lee S.-H., Moon U. (2010). Merged capacitor switching based SAR ADC with highest switching energy-efficiency. Electron. Lett..

[5] Yuan C., Lam Y. (2012). Low-energy and area-efficient tri-level switching scheme for SAR ADC. Electron. Lett..

[6] Verma N., Chandrakasen A. (2007). An ultralow energy 12-bit rate-resolution scalable SAR ADC for wireless sensor nodes. IEEE J. Solid-State Circuits.

[7] Yoshioka M., Ishikawa K., Takayama T., Tsukamoto S. (2010). A 10-b 50-Ms/s 820-μW SAR ADC with on-chip digital calibration. IEEE Trans. Biomed. Circuits Syst..

[8] Lee H.-S., Hodges D.A., Gray P.R. (1984). A self-calibrating 15 bit CMOS A/D converter. IEEE J. Solid-State Circuits.

[9] Wang X., Huang H., Li Q. (2015). Design considerations of ultralow-voltage self-calibrated SAR ADC. IEEE Trans. Circuits Syst. II-Exp. Briefs.

[10] Liu W., Huang P., Chiu Y. (2011). A 12-bit, 45-MS/s, 3-mW redundant successive-approximation-register analog-to-digital converter with digital calibration. IEEE J. Solid-State Circuits.

[11] Um J.Y., Kim Y.-J., Song E.-W., Sim J.-Y., Park H.-J. (2013). A digital-domain calibration of split-capacitor DAC for a differential SAR ADC without additional analog circuits. IEEE Trans. Circuits Syst. I-Reg. Pap..

[12] Chang K.H., Hsieh C.C. A 12b 10 MS/s 18.9 fJ/conversion-step sub-radix-2 SAR ADC. Proceedings of the 2016 International Symposium on VLSI Design, Automation and Test (VLSI-DAT).

[13] Chang D.-J., Kim W., Seo M.-J., Hong H.-K., Ryu S.-T. (2017). Normalized-full-scale-referencing digital-domain linearity calibration for SAR ADC. IEEE Trans. Circuits Syst. I-Reg. Pap..

[14] Harpe P., Zhou C., Bi Y., Meijs N.P., Wang X., Philips K., Dolmans G., Groot H. (2011). A 26 μW 8 bit 10 MS/s asynchronous SAR ADC for low energy radios. IEEE J. Solid-State Circuits.

[15] Harpe P., Cantatore E., Roermund A. An oversampled 12/14b SAR ADC with noise reduction and linearity enhancements achieving up to 79.1 dB SNDR. Proceedings of the 2014 IEEE International Solid-State Circuits Conference Digest of Technical Papers (ISSCC).

[16] van Elzakker M., van Tuijl E., Geraedts P., Schinkel D., Klumperink E., Nauta B. (2010). A 10-bit charge-redistribution ADC consuming 1.9 μW at 1 Ms/s. IEEE J. Solid-State Circuits.

[17] Lei K.-M., Mak P.-I., Martins R.P. (2013). Systematic analysis and cancellation of kickback noise in a dynamic latched comparator. Analog Integr. Circ. Sig. Process.

[18] Razavi B. (2001). Design of Analog CMOS Integrated Circuits.

[19] Wong K.-L.J., Yang C.-K.K. (2004). Offset compensation in comparators with minimum input-referred supply noise. IEEE J. Solid-State Circuits.

[20] Babayan-Mashhadi S., Lotfi R. (2014). Analysis and design of a low-voltage low-power double-tail comparator. IEEE Trans. Very Large Scale Integr. Syst..

[21] Shikata A., Sekimoto R., Kuroda T., Ishikuro H. (2012). A 0.5 V 1.1 MS/sec 6.3 fJ/conversion-step SAR-ADC with tri-level comparator in 40 nm CMOS. IEEE J. Solid-State Circuits.

[22] Ding M., Harpe P., Liu Y.-H., Busze B., Philps K., Groot H. (2017). A 46 μW 13 b 6.4 MS/s SAR ADC with background mismatch and offset calibration. IEEE J. Solid-State Circuits.

[23] Huang H., Ao K., Guo Z., Li Q. A 0.5 V rate-resolution scalable SAR ADC with 63.7dB SFDR. Proceedings of the 2013 IEEE International Symposium on Circuits and Systems (ISCAS2013).

[24] Dessouky M., Kaiser A. (2001). Very low-voltage digital-audio ΔΣ modulator with 88-dB dynamic range using local switch bootstrapping. IEEE J. Solid-State Circuits.

[25] Daly D.C., Chandrakasan A.P. (2009). A 6-bit, 0.2 V to 0.9 V highly digital flash ADC with comparator redundancy. IEEE J. Solid-State Circuits.

[26] Hung G.-Y., Chang S.-J., Liu C.-C., Lin Y.-Z. (2012). A 1-μW 10-bit 200 kS/s SAR ADC with a bypass window for biomedical applications. IEEE J. Solid-State Circuits.

[27] Ha H.S., Lee S.-K., Kim B.S., Park H.-J., Sim J.-Y. (2014). A 0.5-V, 1.47-μW 40-kS/s 13-bit SAR ADC with capacitor error compensation. IEEE Trans. Circuits Syst. II-Exp. Briefs.

[28] Yip M., Chandrakasan A.P. (2013). A resolution-reconfigurable 5-to-10-bit 0.4-to-1 V power scalable SAR ADC for sensor applications. IEEE J. Solid-State Circuits.

[29] Zhang D., Bhide A., Alvandpour A. (2012). A 53-nW 9.1-ENOB 1-kS/s SAR ADC in 0.13 μm CMOS for medical implant devices. IEEE J. Solid-State Circuits.

